# Transvenous dual-chamber pacemaker implantation in patients with persistent left superior vena cava

**DOI:** 10.1186/s12872-019-1082-7

**Published:** 2019-04-29

**Authors:** Teng Li, Qiong Xu, Hong-tao Liao, Dimitrios Asvestas, Konstantinos P. Letsas, Yifu Li

**Affiliations:** 1 Arrhythmia Department, Fuwai Hospital Chinese Academy of Medical Sciences, Shenzhen, 518057 Guangdong China; 2 Cardiovascular Department, Guangdong Cardiovascular Institute, Guangdong General Hospital & Guangdong Academy of Medical Sciences, Guangzhou, 510010 China; 3 Second Department of Cardiology, Laboratory of Cardiac Electrophysiology, “Evangelismos” General Hospital of Athens, Athens, Greece

**Keywords:** Persistent left superior vena cava, Pacemaker, Implantation, Active fixation lead

## Abstract

**Background:**

Persistent left superior vena cava (PLSVC) is a rare congenital vascular anomaly. Permanent pacemaker implantation (PPI) in patients with PLSVC can be challenging because of the venous anomalies. We reported a case series of patients with PLSVC who underwent PPI with double active fixation leads.

**Methods:**

From January 2012 to July 2016, 9 patients (three male and six females, mean age 68 ± 11 years) with PLSVC who received a dual-chamber pacemaker with double active fixation leads were enrolled retrospectively in this observational study. The indications for pacemaker implantation were symptomatic third-degree atrioventricular block in one and sick sinus syndrome in eight patients.

**Results:**

PPI were implanted successfully in all 9 patients. Successful positioning of the ventricular leads at the right ventricular outflow tract (RVOT) septum with a “C” shaped stylet was achieved in 7 patients (77.8%). In the remaining two cases, the ventricular leads were placed in the right ventricular apex and the inferior free wall of the sub-tricuspid annulus. The atrial leads were placed at the lateral wall of the right atrium in all patients. Procedure time and fluoroscopy time were 85.3 ± 11.3 min and 4.5 ± 1.1 min respectively. During a mean follow-up of 4 years, no complications were observed and pacing parameters did not change significantly.

**Conclusion:**

PPI through PLSVC may be technically feasible, safe, and effective. Double active fixation leads may be standard for patients with PLSVC and most of the ventricular leads could be placed at the RVOT septum.

## Background

Persistent left superior vena cava (PLSVC) is a rare congenital vascular anomaly, occurring in 0.3 to 0.5% of individuals in the general population [1]. Transvenous permanent pacemaker implantation (PPI) in patients with PLSVC is challenging because of the complex anatomy. The coronary sinus (CS) may be dilated, which render pacing leads positioning from the left subclavian region difficult, especially the ventricular lead. The literature regarding PPI in patients with PLSVC is sparse and limited to a few case reports [2–8]. The use of active fixation leads with special curved stylet may help in overcoming this technical difficulty [2]. The purpose of this study was to describe the methods and evaluate the efficacy of patients with PLSVC who underwent PPI with double active fixation leads at our institutions.

## Methods

### Study population

From January 2012 to July 2016, 2420 patients underwent PPI with symptomatic atrioventricular block (AVB) or sick sinus syndrome (SSS). Among 2420 cases, only 9 (9/2420, 0.37%) patients underwent PPI were found with PLSVC. 9 patients were enrolled retrospectively in this observational study.

The present study was performed in accordance with the Declaration of Helsinki (2000), and it was approved and supervised by the Ethics Committee of Fuwai Hospital Chinese Academy of Medical Science and Guangdong General Hospital. All patients and relatives were given full explanations of the procedures, and written informed consent was obtained from all subjects.

### Preoperative preparation

All patients received a dual-chamber pacemaker (ADAPTAL ADDRL or SENSIA SEDRL, Medtronic, Minneapolis, MN, USA, or ZEPHYR XLDR 5826, St. Jude Medical, St. Paul, MN, USA) with double active fixation leads. 58-cm bipolar active fixation leads with steroid-eluting electrodes (CAPSUREFIX NOVUS 5076, Medtronic, Minneapolis, MN, USA, or TENDRIL ST 1888TC, St. Jude Medical, St. Paul, MN, USA) were used for both atrial and ventricular pacing in all patients.

PPI was performed during the fasting state under local anesthesia. Prophylactic intravenous antibiotic was administered 30 min prior to the procedure. The procedures were undertaken under strict aseptic precautions with venous access gained by the left axillary vein or the left subclavian vein in all patients. Meanwhile, the pacemaker pocket was performed routinely in the left subclavian region. Venous access for the atrial and the ventricular leads was gained through two separate punctures without any complications.

### Implantation procedure

**Fig. 1:**
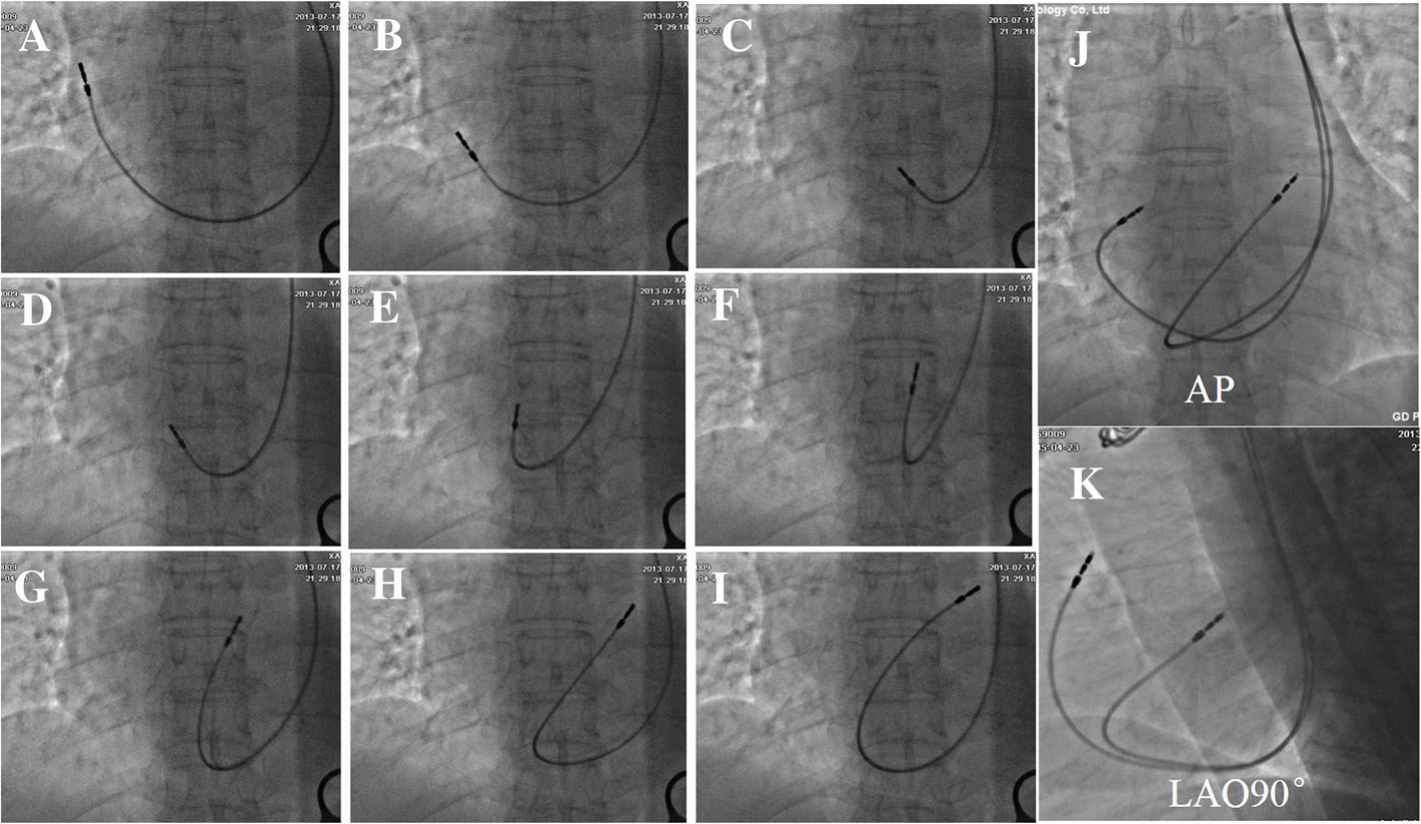
The implantation steps of the ventricular lead with a “C” shaped stylet in one patient (**a-i**). AP view and LAO 90° view of the double active fixation leads implanted via PLSVC (**j-k**). PLSVC = Persistent left superior vena cava; AP = anterior-posterior; LAO = left anterior oblique

Venography was performed to confirm the presence of PLSVC if necessary. The ventricular lead was attempted to be placed in the RVOT septum. Otherwise, the ventricular lead was placed in the right ventricular apex (RVA) region. Both atrial and ventricular pacing leads were advanced into the right atrium with the original soft straight stylet first. Then a “C” shaped stylet or J-shaped stylet was used to introduce the ventricular lead via the tricuspid annulus to the RVOT. And then, the ventricular lead pointed toward the tricuspid annulus with a slight withdrawal and clockwise rotation, so it could be advanced into and placed at the RVOT (Fig. 1). The atrial lead was positioned in the lateral atrial wall or the right atrial appendage (RAA) without using other differently shaped stylets. The final position of the double pacing leads was evaluated during the procedure using fluoroscopic projections including anterior-posterior (AP) view, right anterior oblique (RAO) 30°view, left anterior oblique (LAO) 45°view and left lateral (LL) 90° view. Pacing parameters were obtained at the end of the procedure, including pacing impedance (Ω), pacing threshold (V), P-wave and R-wave amplitude (mV).

### Follow-up

After pacemaker implantation, all patients were followed up every 6 months regularly as outpatients. Pacing parameters including pacing threshold, electrode impedance, P-wave and R-wave amplitude were assessed. Chest X-ray and transthoracic echocardiography were performed during follow-up if necessary.

## Results

Nine patients (6 women and 3 men) with PLSVC who underwent dual-chamber pacemaker implantation using double active fixation leads were included in the study retrospectively. Patients’ characteristics and procedure data were summarized in Table 1. The average age of the 9 patients with PLSVC was 68 ± 11 years (52–77 years). Indications for pacing were symptomatic third-degree atrioventricular (AV) block in one (11%) and sick sinus syndrome in eight patients (89%). One patient was referred for dual-chamber pacemaker implantation due to permanent complete AV block after mitral valve replacement. The pacemaker pocket was performed entirely in the left subclavian region irrespectively of the PLSVC.

**Table 1:** Patients’ baseline and implant data

Patients	9
Age (years)	68 ± 11
Male, n (%)	3 (33.3%)
LVEF (%)	62 ± 4
AVB, n (%)	1 (11.1%)
SSS, n (%)	8 (88.9%)
Ventricular leads position, n (%)	
RVOT septum	7 (77.8%)
RVA	1 (11.1%)
Free wall of TV	1 (11.1%)
Threshold (V)	
RA lead	1.0 V ± 0.3
RV lead	0.9 ± 0.2
Amplitude (mV)	
P-wave	3.2 ± 1.1
R-wave	10.4 ± 2.2
Impedance(Ω)	
RA lead	560 ± 120
RV lead	550 ± 95
Procedure time (min)	85.3 ± 11.3
fluoroscopy time (min)	4.5 ± 1.1

*RA* Right atrium, *RV* Right ventricle, *SSS* Sick sinus syndrome, *AVB* Atrial ventricular block, *RVOT* right ventricular outflow tract, *TV* Tricuspid annulus

**Fig. 2:**
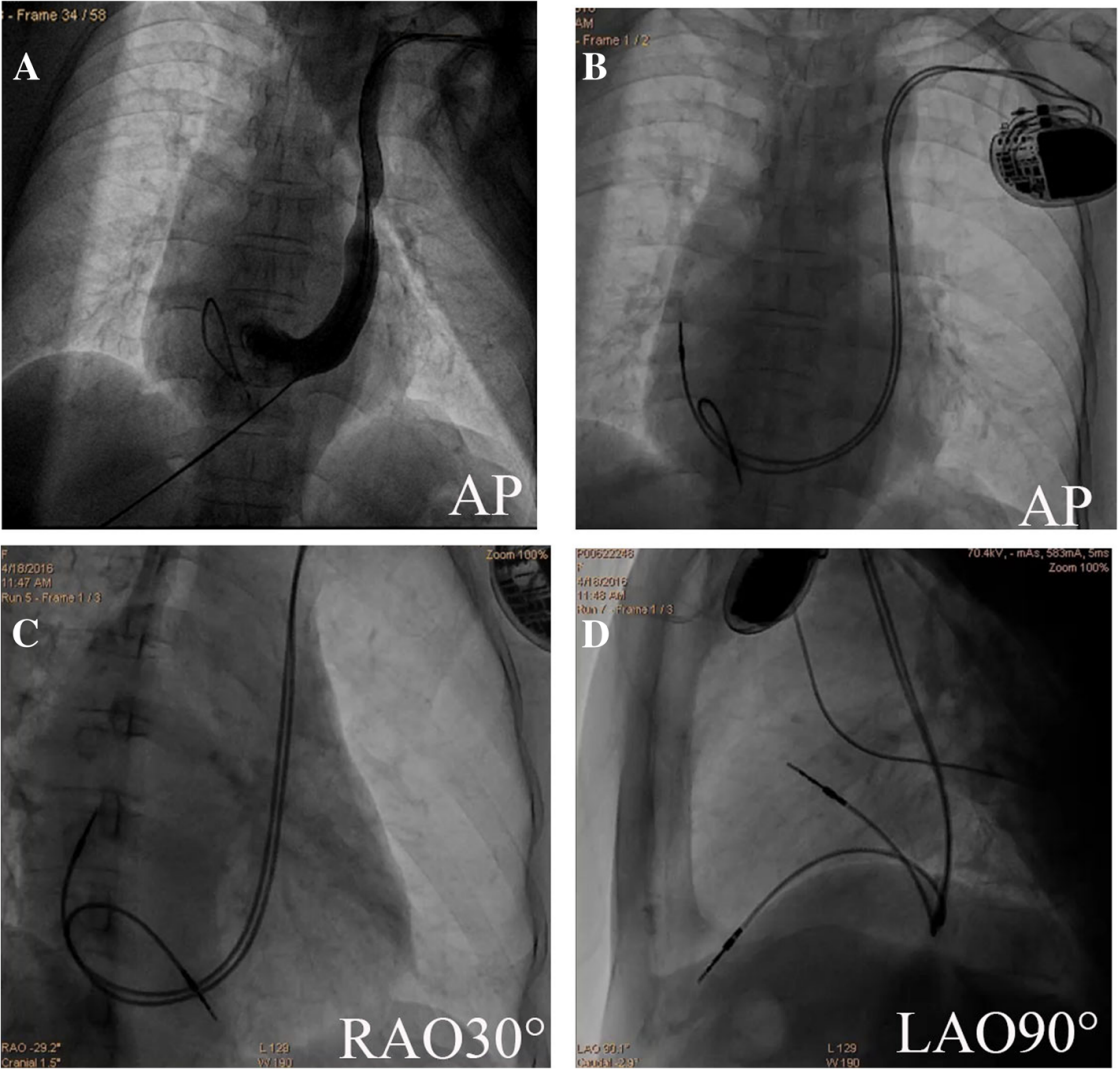
Fluoroscopic views of the atrial and the ventricular leads in another patient with PLSVC at the end of procedure. PLSVC was demonstrated by left subclavian vein angiography in AP view, the temporary pacemaker lead was also placed at the RVA (**a**). The atrial lead was placed at the right lateral wall, and the RV lead was fixed in the RVA (**b**-**d**). PLSVC = Persistent left superior vena cava; RVA = right ventricular apex; AP = anterior-posterior; RAO = right anterior oblique; LAO = left anterior oblique

**Fig. 3:**
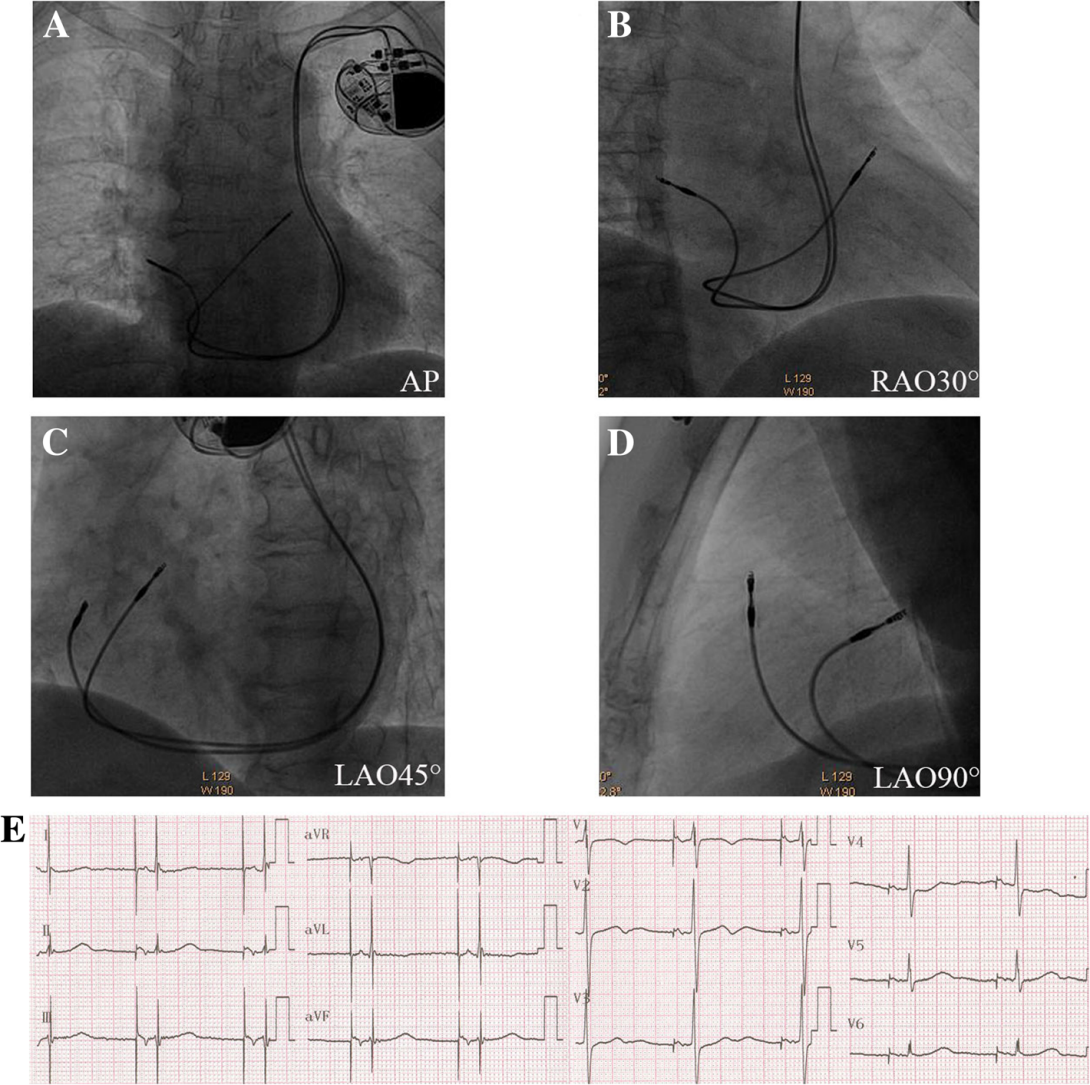
Final fluoroscopic images of Patient 3. The ventricular lead was fixed in high-septal of RVOT in both the views (**a-d**). Twelve-lead surface electrocardiogram recordings after the pacemaker implantation procedure (**e**). In this patient, the ventricular lead was seen pointing towards the RVOT septum (**d**). RVOT = right ventricular outflow tract. Other abbreviations as in Fig. 2

A temporary pacing lead was inserted through the left subclavian vein in 2 patients due to the frequent symptomatic sinus pause prior to the PPI procedure. Venography was also performed to confirm the presence of PLSVC only in these two patients (Fig. 2). The ventricular lead was advanced into the RVOT and placed in the RVOT septum with a “C” shaped stylet easily in 7 patients (Fig. 3). Several attempts failed to introduce and advance the ventricular lead into the RVOT in the remaining 2 patients, not even using differently shaped stylets. For this reason, the ventricular leads were fixed in the RV apical region and the inferior free wall of sub-tricuspid annulus respectively. The atrial leads were placed at the right lateral wall with an original soft straight stylet in all patients (100%).

Total procedure time and fluoroscopy time was 85.3 ± 11.3 min and 4.5 ± 1.1 min respectively. There were no complications during any of the procedures.

### Outcomes during follow-up

Furthermore, no late complications such as lead fracture, lead dislodgement, pericardial tamponade, or chest pain were observed during a mean follow-up of 4 years. In addition, pacing impedance, pacing threshold, P-wave and R-wave amplitude did not change significantly during the follow-up.

## Discussion

Our results demonstrated the clinical safety of PPI through PLSVC, which may be technically feasible and effective. Double active fixation leads appear to be an effective procedure, especially in patients with PLSVC and we recommend this therapeutic approach rather than the choice of the contralateral right-sided implantation.

Active fixation pacing leads are more commonly used, as they are associated with easy fixation, reliability, lower rates of dislodgement and selective pacing, especially with abnormal anatomy. Moreover, active fixation leads offer the convenience of lead extraction. Therefore, double active fixation leads are highly recommended for patients with PLSVC and other abnormal anatomy. Stable lead thresholds were noted even on long-term follow-up that ranged up to 6 years.

The presence of this vascular anomaly is often detected incidentally during placement of cardiovascular implantable electronic devices and deca-polar coronary sinus catheter via left subclavian vein or surgery. PLSVC can also be detected by an experienced ultrasonologist during routine transthoracic echocardiographic examination. In our center, the incidence of PLSVC during device implantation was only found in 0.37% of patients, a similar rate to previous studies [1].

To our knowledge, this is the largest series of implantation of pacemakers in patients with PLSVC. In our study, the ventricular lead of most cases was placed at the septum of RVOT. This position of the ventricular lead seems hemodynamically more profitable than the classic RVA pacing. Furthermore, all PPI was performed in the left subclavian region irrespective of the PLSVC. This was an operator preference. In our center, operators are used to operating on the right subclavian region. At the same time, the absence of right superior vena cava (RSVC) also exists in some patients. Moreover, the vast majority of Chinese patients are right-handed. Although venography prior to the incision of the pocket device may be helpful in identifying the anatomy and the condition of the veins [9], the majority of our patients underwent device implantation safely and successfully without venography. Less experienced surgeons more often choose a right-sided device implantation after confirming a PLSVC by venography, and sometimes the leads can be easily replaced in the right atrium and the right ventricle via the RSVC. However, switching to the right subclavian region may add a new incision and increase the inconvenience for the patients particularly in right-handed patients.

In general, a specific J-shaped stylet designed to facilitate positioning of active fixation ventricular lead into the RV septum was used in patients without PLSVC [10, 11]. Instead, the major challenge for the operator is to advance the ventricular lead into the RVOT via the PLSVC. The existence of an acute angle between the CS ostium and the tricuspid valve makes the advancement and the placement of the lead into the right ventricle technically difficult. Various techniques such as the use of standard, special shaped and right ventricular septal stylets facilitating ventricular lead implantation have been reported [2]. In previous case reports [2, 3, 12], the ventricular leads were fixed only in the RVA rather than the RVOT septum and a right-sided implantation of the ventricular lead is suggested in case of unsuccessful stable ventricular lead position via the PLSVC [3]. In our study, all stable ventricular leads were positioned successfully and safely via a PLSVC. In most of the patients, the ventricular lead was advanced into the RVOT easily with a “C” shaped stylet. Active fixation lead offers the advantage of flexibility of choosing an optimal pacing site in the setting of heart abnormalities and particularly for patients with PLSVC. However, despite several attempts with different shaped stylets, we failed to advance the ventricular leads into the RVOT in two patients which is probably due to the moderate to severe enlargement of the cardiac chamber.

It is well known that the atrial active fixation lead was withdrawn into the right atrium from the right SVC and deployed at or close to the appendage using a J-shaped stylet in patients without PLSVC. Due to enlarged CS ostium, all atrial pacing leads were advanced into the right atrium via PLSVC with an original straight stylet, and the leads were placed at the right lateral wall without specifically shaped stylet in all patients.

Although successful PPI with acceptable pacing parameters were achieved in all patients with no periprocedural complications, optimal pacing or right ventricular mid-septal pacing was much more difficult to achieve than patients without PLSVC. Furthermore, total procedure time and fluoroscopy time in patients PLSVC was significantly longer than those patients without PLSVC. During a mean follow-up of 4 years, no late complications including device malfunction and lead dislodgement were observed. Stable pacing thresholds were noted even on long-term follow-up that ranged up to 6 years. The incidence of complications may be not related to the location of ventricular lead.

### Limitations

There were several limitations in our study. First, it was a small retrospective observational study. Second, we did not further rule out the possibility of complication with other cardiovascular abnormalities. Third, this study was not designed to identify a method in order to improve the technique of the ventricular lead implantation, but to assess the utility of the double active fixation leads in patients with PLSVC. Finally, long steerable sheaths and other special tools which were used for placement of coronary venous leads or His bundle pacing may further facilitate the lead placement. Despite these limitations, our experience suggests that PPI through PLSVC may be technically feasible, and can produce comparable results with those high-expense special tools.

## Conclusion

PPI through PLSVC may be technically feasible, safe, and effective. A venography in patients with PLSVC prior to pacemaker implantation is not necessary. Double active fixation leads may be standard for patients with PLSVC, and most of the ventricular leads could be placed at the RVOT septum.

## Abbreviations

AP: Anterior-posterior
AVB: Atrial ventricular block
CAG: Coronary angiography
CS: Coronary sinus
LAO: Left anterior oblique
LL: Left lateral
LVEF: Left ventricular ejection fraction
PLSVC: Persistent left superior vena cava
PPI: Permanent pacemaker implantation
RA: Right atrium
RAO: Right anterior oblique;
RV: Right ventricle
RV: Right ventricular
RVOT: Right ventricular outflow tract
SSS: Sick sinus syndrome
SVC: Superior vena cava
